# Drug Design in the Exascale Era: A Perspective from Massively Parallel QM/MM Simulations

**DOI:** 10.1021/acs.jcim.3c00557

**Published:** 2023-06-15

**Authors:** Bharath Raghavan, Mirko Paulikat, Katya Ahmad, Lara Callea, Andrea Rizzi, Emiliano Ippoliti, Davide Mandelli, Laura Bonati, Marco De Vivo, Paolo Carloni

**Affiliations:** † Computational Biomedicine, Institute of Advanced Simulations IAS-5/Institute for Neuroscience and Medicine INM-9, Forschungszentrum Jülich GmbH, Jülich 52428, Germany; ‡ Department of Physics, RWTH Aachen University, Aachen 52074, Germany; ¶ Department of Earth and Environmental Sciences, University of Milano-Bicocca, Piazza della Scienza 1, 20126 Milan, Italy; § Atomistic Simulations, Italian Institute of Technology, Genova 16163, Italy; ∥ Molecular Modelling and Drug Discovery, Italian Institute of Technology, Genova 16163, Italy; ⊥ Department of Physics and Universitätsklinikum, RWTH Aachen University, Aachen 52074, Germany

## Abstract

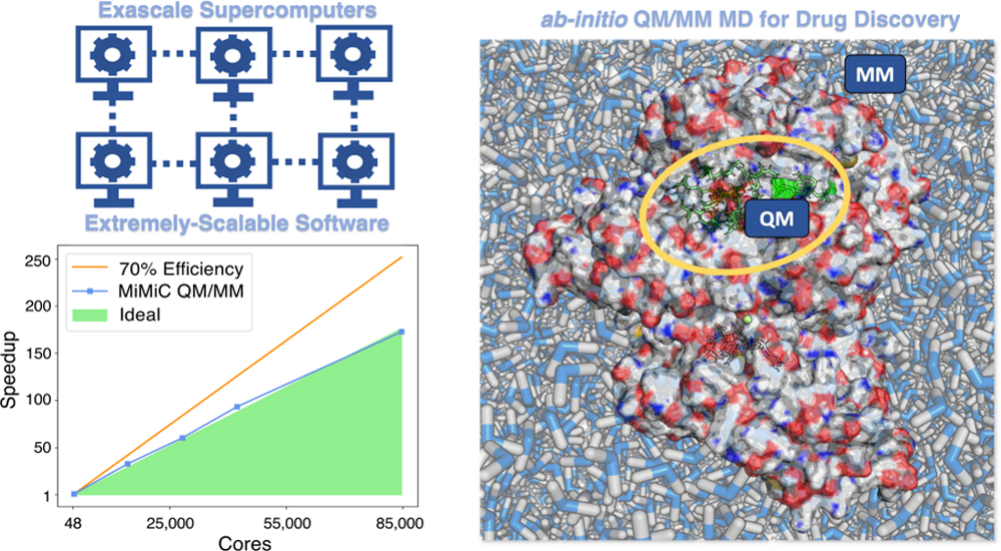

The initial phases of drug discovery – *in silico* drug design – could benefit from first principle Quantum Mechanics/Molecular Mechanics (QM/MM) molecular dynamics (MD) simulations in explicit solvent, yet many applications are currently limited by the short time scales that this approach can cover. Developing scalable first principle QM/MM MD interfaces fully exploiting current exascale machines – so far an unmet and crucial goal – will help overcome this problem, opening the way to the study of the thermodynamics and kinetics of ligand binding to protein with first principle accuracy. Here, taking two relevant case studies involving the interactions of ligands with rather large enzymes, we showcase the use of our recently developed massively scalable Multiscale Modeling in Computational Chemistry (MiMiC) QM/MM framework (currently using DFT to describe the QM region) to investigate reactions and ligand binding in enzymes of pharmacological relevance. We also demonstrate for the first time strong scaling of MiMiC-QM/MM MD simulations with parallel efficiency of ∼70% up to >80,000 cores. Thus, among many others, the MiMiC interface represents a promising candidate toward exascale applications by combining machine learning with statistical mechanics based algorithms tailored for exascale supercomputers.

## Introduction

In the past decade, first-principle Quantum Mechanics/Molecular Mechanics (QM/MM) molecular dynamics (MD) simulations in explicit solvent proved to be a powerful tool to investigate biochemical processes where the electronic degrees of freedom play a major role.^1,2^ In this approach, the region of interest (e.g., the active site of an enzyme) is treated at the QM level, while the rest is described by classical force fields.^3^ The choice of the QM level of theory is generally a compromise between the accuracy required by the problem at hand and the associated computational burden. In this respect, nonempirical density functional theory (DFT) is a rather general (and relatively accurate) approach, and it comes at a far minor computational cost than wave function-based methods including electronic correlation.^4^ As such, DFT-based QM/MM MD simulations are nowadays the method of choice in many state of the art *in silico* studies of biochemical processes, including enzymatic reactions,^5−16^ transition metals binding to proteins,^17−20^ proton transfer,^21−24^ and photophysical processes.^25−28^ Applications to drug design, on the other hand, have not been sufficiently explored, apart from notable exceptions.^29^*Static* DFT QM/MM calculations have been already shown to be very useful, by explaining drug action and by informing about routes for structure based drug design.^30−34^ Accessing the *dynamics* of enzymatic reactions at the DFT level will push the boundaries of pharmacological applications beyond the current state of the art,^12^ extending in particular the domain of application of QM/MM approaches to most metalloenzymes (more than 30% of all proteins^35^). Long time scale DFT QM/MM MD can help in describing the flexibility and the dynamics of complex enzymes, which may be crucial for their function,^36−40^ and in predicting accurate catalytic rates (*k*_cat_) and transition states. The latter represent essential knowledge for the design of transition state analogs,^41,42^ widely considered to be superior to substrate analogs.^43^ First principle QM/MM MD simulations can also serve as a stepping stone toward accurate predictions of ligand binding free energies^44−46^ and residence times (*k*_off_^–1^),^47,48^ very important parameters to assess drug efficiency.^49−51^ However, DFT QM/MM MD comes at a much larger computational cost than static and semiempirical calculations. As a result, the accessible time scales currently reach a few hundreds of ps in state of the art DFT QM/MM simulations including ∼10^2^ QM atoms,^52−54^ severely limiting the statistical accuracy. This is the main bottleneck hindering the widespread utilization of this method for pharmacology, in both academia and industry.

The current exascale revolution in high performance computing presents an exciting opportunity for the DFT QM/MM community to transcend these limitations.^55^ Reaching the exascale requires DFT QM/MM interfaces to scale effectively and take maximum advantage of the large number of networked CPU and GPU cores provided by modern supercomputers. Despite the many efficient DFT QM/MM software available,^56−67^ to the best of our knowledge, scarce information can be found in the literature regarding their strong scaling in pure QM/MM MD applications. In this respect, the Multiscale Modeling in Computational Chemistry (MiMiC) QM/MM framework^68,69^ that couples CPMD^70^ (QM) and GROMACS^71^ (MM) represents a notable exception. MiMiC has been recently developed within a European collaboration, including some of the authors. As we demonstrate in this work, the current version of MiMiC can scale over tens of thousands of processes in a single QM/MM MD run of large enzymes at the B3LYP level of theory. As such, we believe that it is well posed to break the limits of currently achievable time scales in applications to pharmacology. Recent trends in computational chemistry let us envision that this will occur via a clever combination with novel statistical mechanics based algorithms and machine learning techniques. So far, QM codes have not been able to scale efficiently on GPU-equipped distributed architectures. Machine learning methods have already shown to make excellent utilization of GPU resources and could be excellent candidates to push DFT QM/MM MD into the exascale regime.^72,73^

Here, after summarizing some salient aspects of the MiMiC-QM/MM interface and demonstrating its scalability, we present applications of the code to systems of pharmacological relevance, from enzymatic reactions for the prediction of the transition state to inhibitor-enzyme binding toward the investigation of *k*_off_ values. We close by giving our perspective about QM/MM MD simulations for drug design in the exascale era.

## The MiMiC Framework

The MiMiC framework provides a general platform that enables the implementation of multiscale simulation methods through coupling of multiple external programs.^68^ Since its inception, MiMiC has been designed for massively parallel applications. With this in mind, a multiple-program multiple-data model has been adopted, where the external programs are allowed to run simultaneously on independent computing resources while exploiting their existing parallelization strategies. Specifically, MiMiC consists of two libraries: (i) the main MiMiC library,^74^ which provides optimized routines for fast computation of the interactions between different subsystems, and (ii) the MiMiC communication library (MCL),^75^ a lightweight communication library that is used to exchange information between the main MiMiC library and the external programs. Adding a new external program to the MiMiC framework requires a relatively small effort that consists in implementing an MCL-based interface and, if needed, extending the main MiMiC library to support the computation of new interaction terms. Overall, these features make MiMiC a highly flexible and efficient framework for multiscale simulations.

MiMiC currently allows performing QM/MM simulations at the DFT level of theory within an electrostatic embedding scheme^76^ via coupling to the CPMD^77^ and GROMACS^71,78^ codes serving as the QM and MM subprograms, respectively. Preparing input files for MiMiC-QM/MM simulations involves preparing separate input files for both the MM and QM software. This is made easy by the MiMiCPy python library.^79^

In our QM/MM MD simulation, the GROMACS and CPMD software run concurrently on different sets of computing nodes and communicate through the CommLib library. CPMD works as the main MD driver to propagate the equations of motion, and it is also linked to the MiMiC library for the computation of the QM-MM electrostatic interactions. As a result, two sets of calculations – MM and QM plus QM-MM – are carried out in parallel on independent sets of computing nodes, with GROMACS and CPMD, respectively, running continuously for the whole duration of the simulation. This multiple program multiple data approach has several advantages, the first being efficiency. It enables one to exploit all the native parallelization strategies implemented in both codes, it keeps communication between codes to a minimum, and it avoids additional overhead that affects other types of QM/MM implementations that are based on writing/reading restart files on disk at each time step.

Given that (i) the QM part of the computation is by far the most expensive, (ii) the MM computation occurs in parallel, and (iii) the communication overhead via CommLib is negligible, the scaling of MiMiC-QM/MM MD simulations is determined solely by CPMD, which was chosen as the QM layer precisely because of its excellent scaling. Indeed, one of the main motivations to develop the MiMiC framework has been to improve upon the old CPMD-based QM/MM interface, which suffered from most of the above-mentioned issues, killing the excellent CPMD scaling performance.

Thanks to CPMD’s very efficient use of standard CPU nodes, this implementation has already displayed strong scaling well beyond ten thousand cores while maintaining an overall parallel efficiency above 70% in a single QM/MM MD simulation of an antiporter protein embedded in a solvated lipid bilayer.^69^ More recently, MiMiC-QM/MM simulations have been used to investigate the thermodynamics of transport processes in membrane channels and transporters,^21,22^ demonstrating the possibility of routine subns QM/MM MD runs of rather large systems.

**Figure 1:**
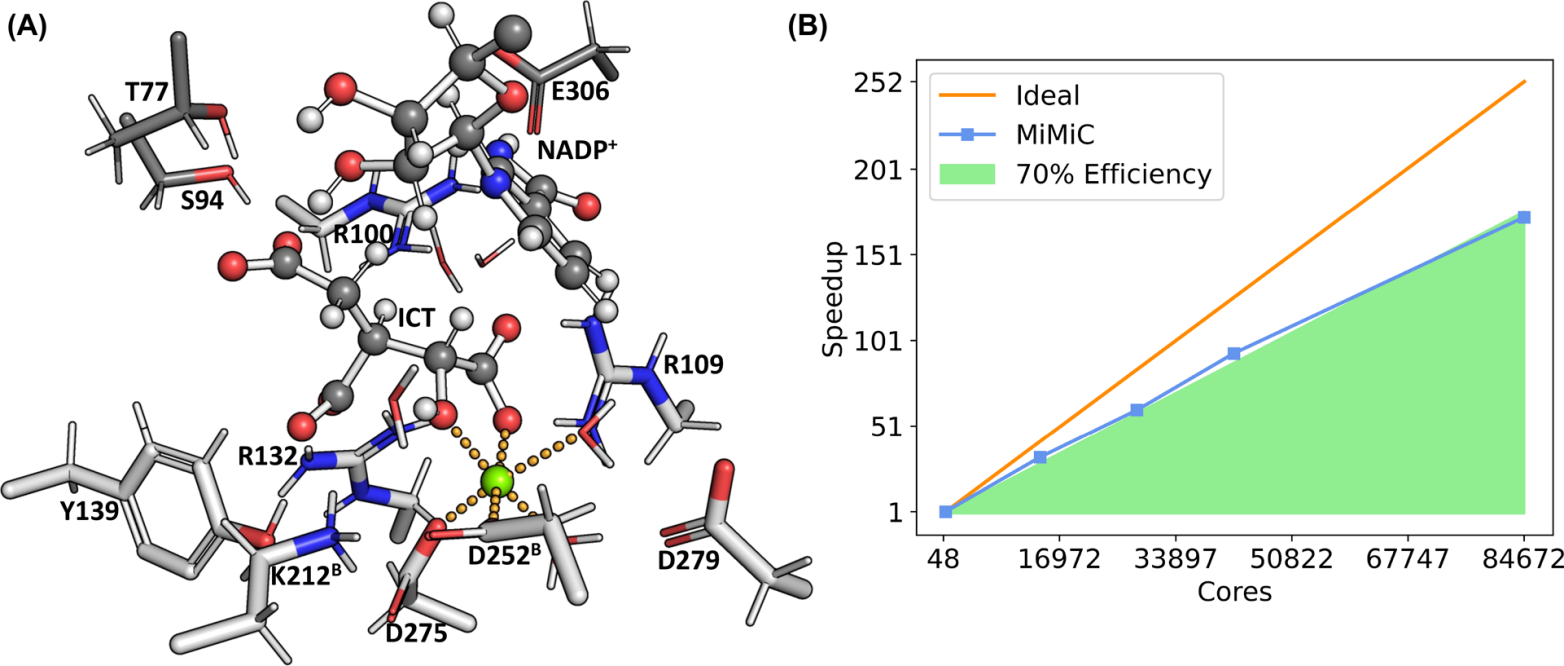
(A) Representation of the Michaelis complex of the IDH1 active site from classical MD simulations. ICT, part of the NADP^+^ pictured, and all residues in light gray are placed in the QM region in our MiMiC-QM/MM simulations. (B) Strong scaling of MiMiC-based DFT QM/MM MD simulations at the B3LYP level of theory of IDH1 as a function of the number of cores assigned to CPMD. In all simulations, we assigned one node (48 cores) to GROMACS. The speedup is provided in terms of the CPU time required for one MD step, normalized with respect to the reference run done on seven nodes. All simulations have been performed on the JUWELS cluster.^80^

Here, for the first time, we demonstrate extreme scalability of MiMiC-QM/MM MD for the investigation of enzymatic reactions, considering the case study of human Isocitrate Dehydrogenase-1 (IDH1). The solvated protein consists of 130,828 atoms in total, and 142 atoms from the active site were assigned to the QM region with a box size of 46.0 au × 46.0 au × 46.0 au. Figure 1 shows the Michaelis complex of the enzyme, as obtained from preliminary classical MD simulations, together with the results of strong scaling benchmarks performed using the B3LYP^81^ functional, showing parallel efficiency ∼70% up to 84,672 cores (1764 JUWELS nodes), and achieving a performance of 0.74 ps/day. Running at this configuration would require around 2.7 Mcore-h/ps. Subsequent node configurations allowing for load balancing in CPMD exceed the size of the JUWELS cluster. This prevented us from further testing the scaling and achieving better B3LYP performance with MiMiC. This underscores the potential of exascale computers that could push further the scalability of QM/MM MD of biological systems. Using the cheaper BLYP functional,^81^ MiMiC-QM/MM MD simulations scale efficiently up to 5,184 cores, achieving a performance of 5.4 ps/day (see the Supporting Information). Running at this configuration would require around 0.02 Mcore-hours/ps.

We also performed scaling benchmarks for the inhibitor-enzyme complex formed by the p38α enzyme and ligand **2g**, which served as our second case study in this work. This system was smaller, with only the 46 atoms of the ligand included in the QM region. MiMiC-QM/MM MD at the BLYP level achieved performance of ≈21 ps/day on 384 cores of the JUWELS cluster module. Running at this configuration would require around 439 core-hours/ps. Benchmarks at the B3LYP level, on the other hand, showed parallel efficiency above 70% up to 12,288 cores with a performance of ≈4.8 ps/day (see the Supporting Information for details). Running at this configuration would require around 0.06 Mcore-hours/ps.

## Investigation of an Enzymatic Reaction: The Case of Human Isocitrate Dehydrogenase-1

The NADP^+^-dependent IDH1 enzyme catalyzes the conversion of isocitrate (ICT) to α-ketoglutarate (α -KG) in the Krebs cycle.^82^ The enzyme is a homodimer and it requires both the NADP^+^ cofactor and a Mg(II) ion:^83,84^
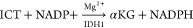


Mutations at the Arg132 position in the active site impart the ability to convert αKG to 2-hydroxyglutarate (2-HG),^85^ an oncometabolite that promotes stemness in human cells and inhibits DNA demethylases.^86,87^ Such variants are involved in the progress of low-grade glioma, glioblastoma, and acute myeloid leukemia (AML).^88^ Describing wild-type and variant IDH1 reaction mechanisms may help design transition-state analogs that act as selective inhibitors of mutant IDH1 and are able to interfere with such diseases.

**Figure 2:**
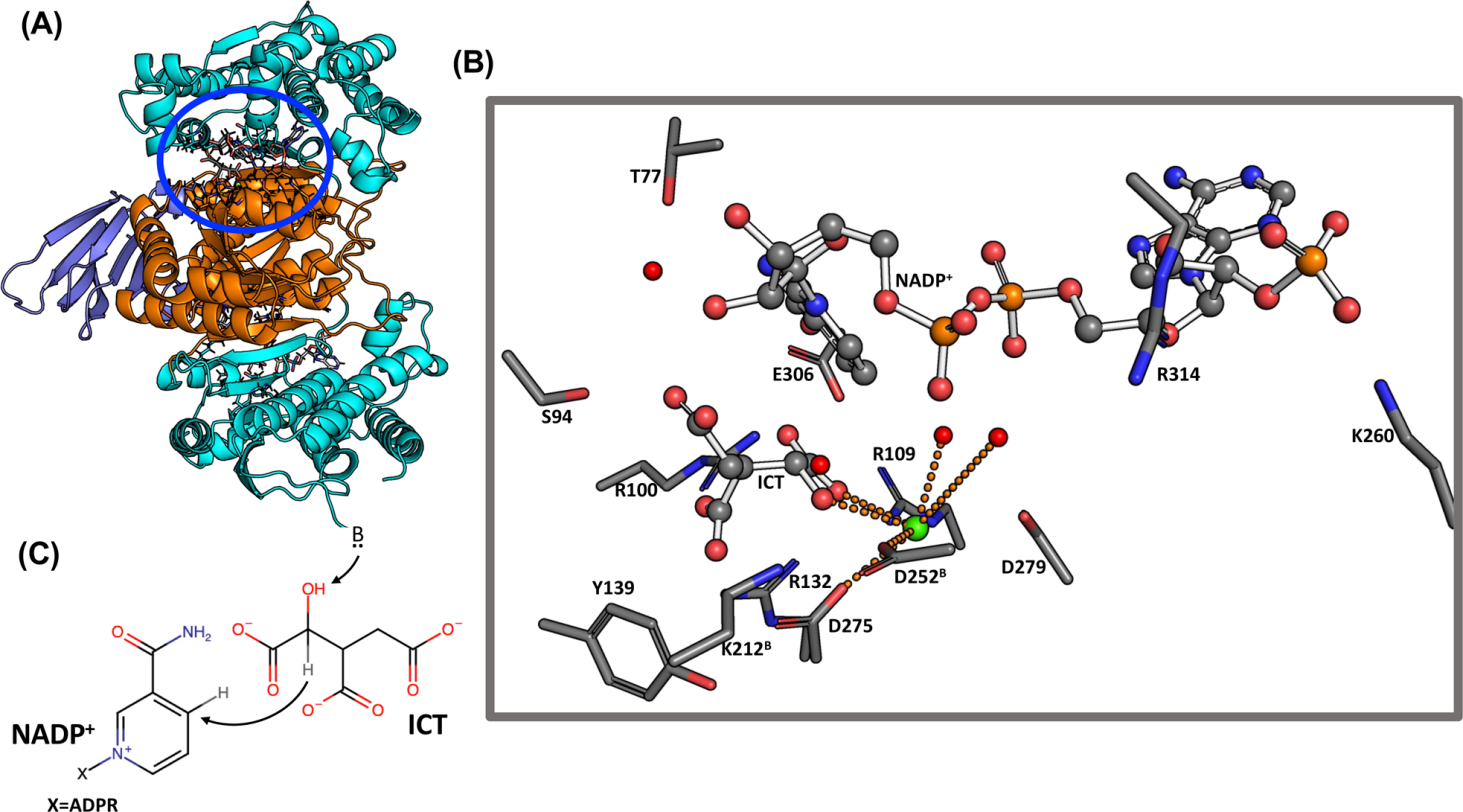
(A) Cartoon representation of the IDH1 enzyme with ICT and NADP^+^. (B) Representation of the IDH1 active site from the X-ray structure. ICT and NADP^+^ are shown in a ball-and-stick representation, while the protein residues are shown as sticks. Crystal waters are shown as red spheres. The Mg^2+^ ion (shown in green) coordination interactions are shown as orange dotted lines. (C) Proposed first step of the IDH1 enzymatic catalysis.^89^

The reaction of wild-type IDH1 has been proposed to occur in a multistep way.^89^ The first step comprises two substeps, corresponding to the deprotonation of the C_α_ hydroxyl of ICT to Oxalosuccinate (OXS) initiated by a base and followed by reduction of NADP^+^ to NADPH by accepting the C_α_ hydride of ICT (see Figure 2C). Notably, the base has not yet been definitively identified. This step is followed by the loss of the C_β_ carboxylate of OXS to give enolate, with the protonation of this enolate resulting in α-ketoglutarate. The X-ray structure of the protein in complex with ICT and NADP^+^ (Figure 2A) shows that each of the two monomers consists of a large domain, a small domain, and a clasp domain. Two active sites include residues from both monomers, held together in the dimer by the clasp domain.^82^ [Here, residues from the second subunit are labeled by the superscript B, while those from the first subunit are left unmarked.] The α-carboxylate group of ICT forms a direct H-bond with Arg100 and Arg109. Lys212^B^, Arg132, and Tyr139 interact with the β-carboxylate group of ICT through H-bonds. Thr77 (through a water molecule), Ser94, and the NADP^+^ ribose interact with the γ-carboxylate of ICT, while Glu306 forms an H-bond with the NADP^+^ ring. These interactions anchor the NADP^+^ nicotinamide ring close to ICT. The phosphate group of the ribose ring carrying the adenine moiety is held in the active site by interactions with Arg314 and Lys260. The Mg^2+^ ion coordination polyhedron consists of the α-carboxylate group of ICT, the α-alcohol of ICT, Asp275, Asp252^B^, and two water molecules. A third water molecule forms an H-bond with Asp252^B^ and with the α-alcohol of ICT. Because of this interaction, Hurley et al.^90^ suggested that Asp252^B^ is the base in the first step of the catalysis. Grodsky et al. proposed instead that this role was taken by Asp279, based on the finding that the activity of IDH1 with Asp252^B^ mutated to Asn is similar to that of the wild-type.^91^ Later, studies showing that IDH1 with Lys212^B^ mutated to Arg, Gln, and Tyr exhibited lower activity, allowing for the suggestion that Lys212^B^ in its deprotonated configuration could be the key basic residue.^92^ Classical MD of the protein in which Lys212^B^ was either protonated or deprotonated, along with static QM/MM calculations corroborated this suggestion, showing that the activation free energy of the NADP^+^ reduction step is larger when Asp279 is the initiator base (21.4 kcal/mol) than when deprotonated Lys212^B^ is the base (13.4 kcal/mol).^93^ [These calculations used a two-layered ONIOM model at the B3LYP/6-31G(d) level of theory with entropic effects included via harmonic approximation.] This step contributes significantly to the determination of the rate of reaction, and the latter pathway agrees fairly well with the experimentally observed *k*_cat_ value of ≈16 kcal/mol.^94^

Here, we apply classical and MiMiC-QM/MM MD to study the conversion of ICT to OXS and the subsequent reduction of NADP^+^ by IDH1 with Lys212^B^ in its protonated configuration [Details of the simulation setups and additional analysis are reported in the Supporting Information.]

### Classical MD

**Figure 3:**
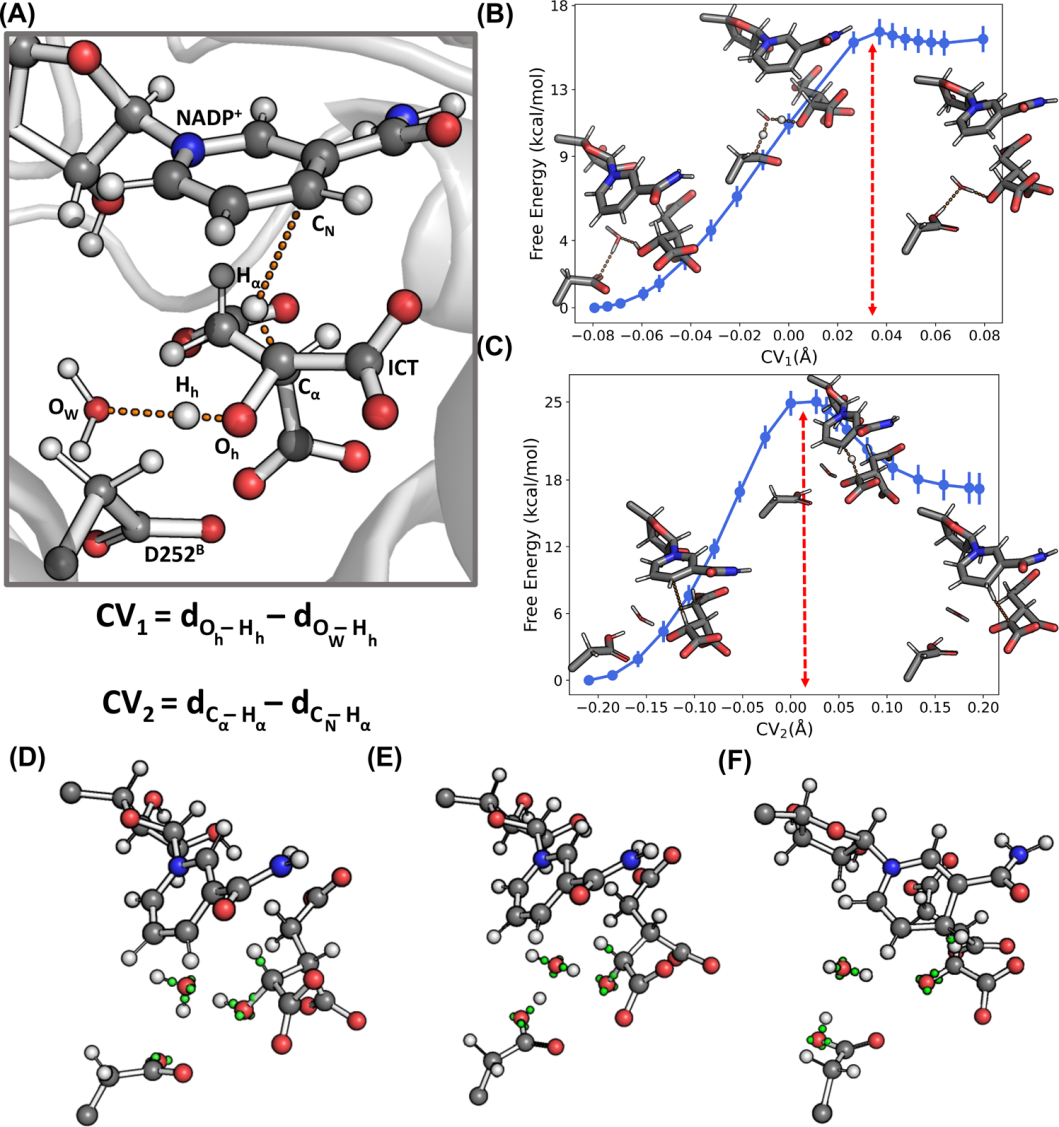
(A) Definition of the collective variables CV_1,2_ used for thermodynamic integration. (B) Free energy of the ICT to OXS conversion with respect to CV_1_. (C) Free energy of the NADP^+^ reduction with respect to CV_2_. Wannier centers depicted in green for select bonds at (D) CV_1_ ≈ −0.08 Å, (E) CV_1_ ≈ 0.08 Å or CV_2_ = 0 Å, and (F) CV_2_ ≈ 0.2 Å.

Figure 1A shows the structure of the Michaelis complex of IDH1 with protonated Lys212^B^ as obtained from our simulations: the network of interaction involving the Mg^2+^ ion, ICT, NADP^+^, and the protein residues in the active site are qualitatively very similar to the X-ray structure described previously. This includes the water molecule forming an H-bond with Asp252^B^ and the α-alcohol of ICT. A significant difference is that Arg100 has moved away from the α-carboxylate of ICT, establishing a water-mediated interaction, in agreement with similar observations by Neves et al.^93^ Thr77, on the other hand, moves closer to and interacts directly with the γ-carboxylate of ICT compared to the crystal structure. Asp252^B^ is well positioned to abstract a proton from the C_α_ hydroxyl of ICT through an H-bonded water molecule (see Figure 3A). This allows us to suggest that Asp252^B^ is a potential candidate base. Asp279, on the other hand, interacts with Mg^2+^ through one of the water molecules coordinating with the metal ion. This mediated interaction moves the residue farther away from the ICT alcohol. Thus, based on our model, we conclude that Asp279 is not a likely candidate for acting as a basis in the first step of the reaction.

### MiMiC-QM/MM MD

The free energy associated with the conversion of ICT to OXS mediated by Asp252^B^, followed by the reduction of the NADP^+^ ring, is investigated via thermodynamic integration.^95,96^ The conversion of ICT to OXS mediated by the Asp252^B^-water pair is described using as the collective variable (CV) the difference CV_1_ = d_O_h_–H_h__ – d_H_h_–O_W__ between the distances of the proton from the two relevant oxygen atoms, while the reduction of NADP^+^ to NADPH is described using the difference CV_2_ = d_C_α_–H_α__ – d_H_α_–C_N__ (see Figure 3A). The obtained free energy profiles along CV_1_ and CV_2_ are reported in Figure 3B and C, where the insets show representative starting, transition state, and final configurations. Simulations were performed at the BLYP level. A cumulative 39 ps of MD were performed, which were obtained in the span of 1 week (see the Supporting Information for more details on the steps used in thermodynamic integration).

To investigate the nature of the bond breaking formation in the QM region during the course of the reaction (see Figure 3D–F), we make use of the Wannier center analysis. In the reactant state (CV_1_ ≈ −0.08 Å), the C_α_–O_h_ bond length is equal to 1.6 Å, with a Wannier center located at ≈1.0 Å from C_α_, indicating a single bond character. At CV_1_ = 0 Å, close to the putative transition state, the water molecule exists as a hydronium ion stabilized by Asp252^B^. In this configuration, O_W_ interacts with H_h_, while one of the hydrogen atoms bound to O_W_ interacts with the Asp252^B^ side chain. The Wannier center along the O_h_–H_h_ bond is located farther away from H_h_ than in the reactant state by ≈0.2 Å, indicating an increasingly higher polar character of the bond and the transfer of a proton to O_W_. This Wannier center is more closely associated with O_h_, indicating a developing negative charge on it. In the final product (CV_1_ ≈ 0.08), the C_α_–O_h_ bond length decreases to ≈1.3 Å, and the Wannier center along the bond is ≈0.8 Å away from C_α_. Furthermore, Asp252^B^ is protonated, and the ICT C_α_ hydroxyl group is deprotonated with a negatively charged O_h_, due to the extra third Wannier center associated with it.

Starting from the product state of the first substep, we calculated the free energy change with increasing CV_2_. In the reactant state (CV_1_ ≈ −0.2 Å), the Wannier center along the C_α_–H_α_ bond is ≈0.7 Å from C_α_ and ≈3.5 Å from C_N_ of the NADP^+^ ring. At the transition state (CV_2_ ≈ 0 Å), the hydride transfer of H_α_ to C_N_ takes place. The Wannier center along the C_α_–H_α_ bond is now ≈1.3 Å from C_α_ and ≈1.5 Å from C_N_. Furthermore, the third Wannier center associated with O_h_ from the product of the previous step has now moved closer to C_α_ (from ≈1.5 Å to ≈1.1 Å) and more along the C_α_–O_h_ bond. This, together with the fact that the C_α_–O_h_ bond length reduces to 1.3 Å, indicates the emergence of a partial double bond character along the C_α_–O_h_ bond. At the product (CV_2_ ≈ 0.2 Å), this extra Wannier center moves to ≈0.8 Å from C_α_. This results in two Wannier centers along the C_α_–O_h_ bond and indicates the establishment of a full double bond, i.e., the formation of a ketone. The Wannier center along the C_α_–H_α_ bond moves ≈3.4 Å away from C_α_, with this Wannier center falling along the newly formed C_N_–H_α_ bond. The hydride transfer of H_α_ to the NADP^+^ ring is complete.

The free energy barriers obtained for the two steps are ≈16 and ≈24 kcal/mol, respectively (Table 1). These values are not too dissimilar from those of the Asp279 pathway and both are significantly higher than the relevant barrier of 13.4 kcal/mol of the Lys212^B^ pathway. Overall, our results thus support the conclusion of ref. (93) indicating the deprotonated Lys212^B^ as the residue that is more likely acting as the base in the first step of the catalytic process.

**Table 1:** Free Energies (in kcal/mol) Associated with the First Step of the IDH1 Catalysis, for Various Base Residues as Initiators of the Reaction^a^

	Lys212^B^	Asp279	Asp252^B^
Deprotonation of ICT	1.5	12.2	16.6 (±0.7)
Reduction of NADP^+^	13.4	21.4	24.0 (±1.6)

a The Helmholtz free energy for Asp252^B^ as the base is from this work, while the Gibbs free energies for the pathways with Lys212^B^ and Asp279 as the base are from ref (93).

## Investigation of Drug/Enzyme Interactions: The Case of **2g** Binding to p38α Mitogen-Activated Protein Kinase

**Figure 4:**
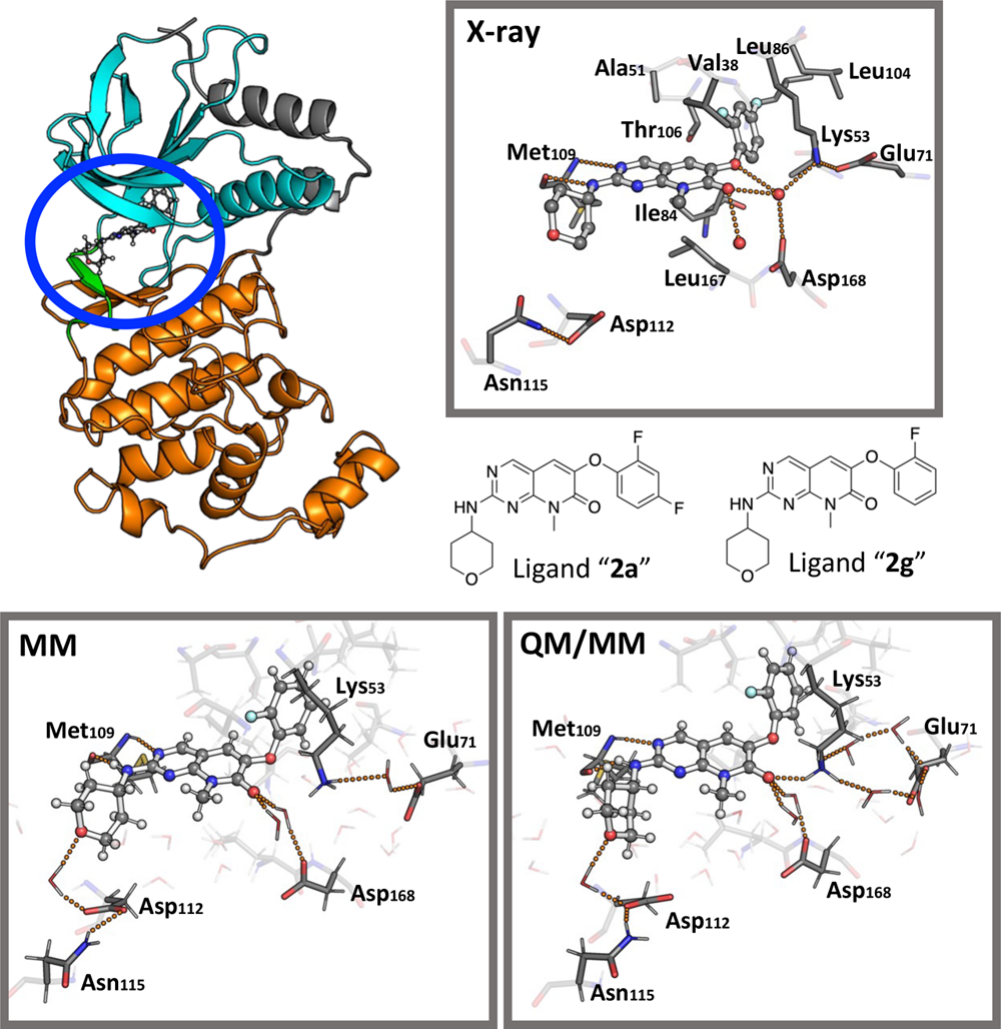
Comparison of X-ray, MM, and MiMiC structures. Upper left panel: Cartoon representation of the p38α MAPK enzyme in complex with a ligand. The N-lobe (cyan) and the C-lobe (orange) of the enzyme are connected via a hinge region (green). The ligand binding pocket is located in between the lobes (blue ellipsis). Upper right panel: Representation of the enzyme binding pocket from the X-ray structure (PDB code: 3FLN).^102^ Ligand **2a** is shown in a ball-and-stick representation, while the protein residues are shown as sticks. Crystal water molecules are shown as red spheres. H-bond interactions are shown as orange dotted lines. The structural formula of ligands **2a** (X-ray) and **2g** (MM and MiMiC simulations) is shown at the bottom of that panel. Lower left panel: Representative snapshot of the binding pocket from the classical MD simulation. Lower right panel: Representative snapshot of the binding pocket from MiMiC simulations.

An accurate description of ligand/enzyme interactions is mandatory to obtain quantitative insights that can guide drug screening and drug design. Indeed, biomolecular force field-based estimates of the drug’s residence time – a key parameter to assess a drug’s efficacy^49−51^ – show a large degree of variations, also depending on the enhanced sampling technique adopted.^48^ Furthermore, static DFT QM/MM calculations directly suggested that limitations of current force fields, which cannot describe charge redistribution of the ligand during the unbinding processes, can contribute to this uncertainty.^47^ DFT QM/MM *molecular dynamics* could be an excellent tool to include these variable charge distributions as such effects are inherently incorporated in this first-principle MD and exascale computers, combined with the power of parallel programming, may help overcome the time scale limitation that currently hampers such applications. Since at the moment a fully QM/MM MD investigation of residence times is out of reach, as a first step toward this very ambitious goal, here we investigate for the first time substrate binding in a pharmacologically relevant enzyme by DFT QM/MM MD using MiMiC to analyze in detail the most important interactions and the dynamics of the bound state. We focus on the p38α enzyme, a member of the mitogen-activated protein kinase (MAPK) family,^97^ in complex with the ligand **2g**. This is a serine/threonine kinase that controls cytokine biosynthesis, and it is involved in the initiation of chronic inflammation processes and development of cancer, heart disease, and many other diseases.^98−101^ It adopts a typical kinase fold, including the N-terminal lobe and C-terminal lobe that are connected via a hinge region (see Figure 4). The catalytic site of the protein is placed between the two lobes, where ATP molecules can bind. The binding of **2g** (Figure 4) is studied based on the X-ray structure complex with its close analogue **2a** (Figure 4, PDB code: 3FLN).^102^ [The ligand names are adopted from ref (102). The IUPAC names are given in the Supporting Information.] The solvated **2g**/p38α complex was obtained by 500 ns-long MD followed by 100 ps-long QM/MM MD simulation at 300 K at the BLYP level.

In the X-ray structure, the phenoxy moiety forms hydrophobic interactions with the residues Ala51, Val38, Leu86, Leu104, Ile84, Thr106, and Leu167 (see Table S2 in the Supporting Information). The pyrimidine N3 atom and the amino group interact instead with the Met109 backbone unit (see Table S2 in the Supporting Information). The rest of the molecule is solvent-exposed.

In the simulations, the mode of binding of the ligand is the same (Figure 4 and Table S2 in the Supporting Information). However, the pyridone oxygen atom interacts at times with Lys53 (*d*(O_2*g*_ ··· H_Lys53_) ≈ 3 Å) because of a water-induced interruption of the Lys53-Glu71 salt bridge (Figure 4), and the tetrahydropyranyl oxygen atom forms a water mediated H-bond with Asp112. [These residues play no role for **2a** binding in the X-ray structure of the **2a**/p38α complex.] This decisive role of water has also been observed in a recent MD study of the p38α MAPK enzyme in complex with different ligands.^103^ In our MiMiC simulations, the first H-bond emerging from the MD simulations becomes persistent (*d*(O_2*g*_···H_Lys53_ ≈ 2.0 ± 0.2 Å (see Figure 4), while the second is retained, although the water molecule mediating the interaction is exchanged within the solvent. The second coordinated water molecule retains its position during the whole simulation and mediates the interaction between the pyridone oxygen atom and the Asp168 residue.

**Figure 5:**
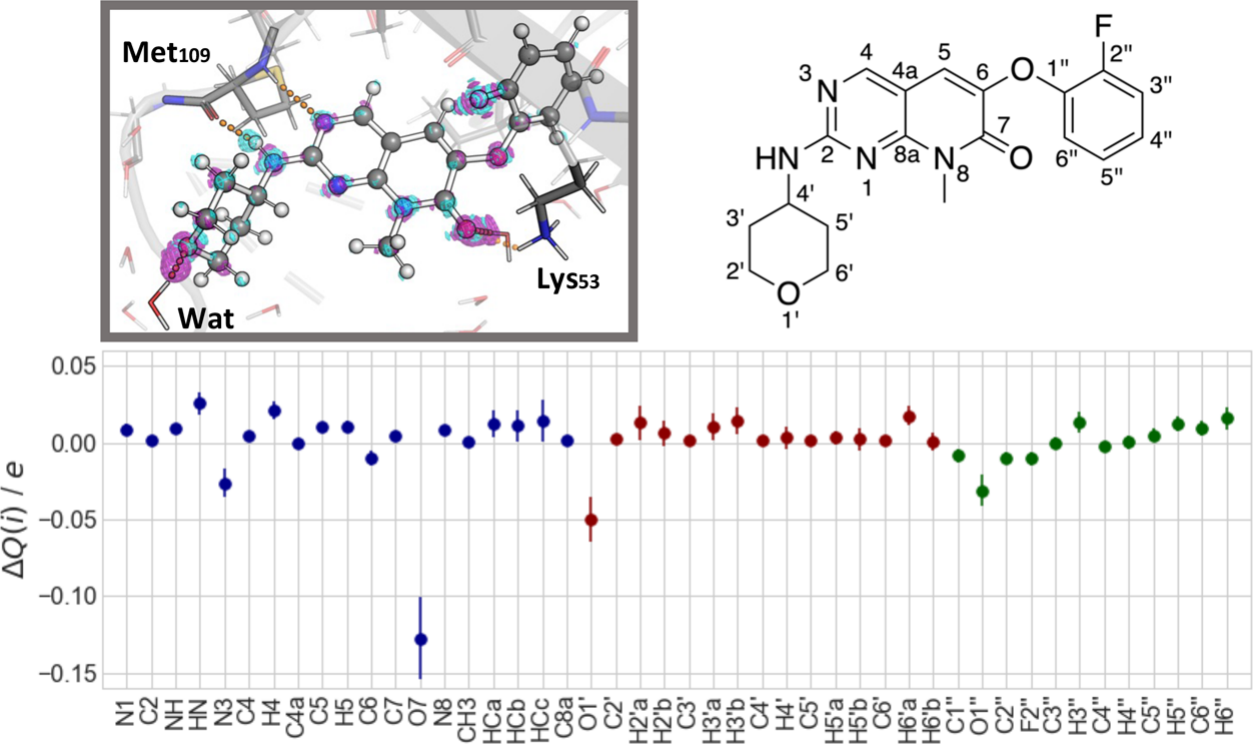
Electronic polarization analysis of the ligand upon passing from the vacuum to the enzyme-bound state. Top left panel: Difference density map for a representative QM/MM snapshot. The difference density is shown as isomesh with a contour level of 0.002 and −0.002 *e* Å^–3^ for increased (magenta) and decreased (cyan) electronic densities, respectively. Top right panel: Structural formula of ligand **2g** indicating the atom numbering. Bottom panel: Averaged change in the atomic partial charges *ΔQ*(*i*) for each ligand’s atom. The data are grouped into the pyridopyrimidone (blue), tetrahydropyranyl (red), and phenoxy (green) moieties.

The effect of electronic polarization of the ligand is investigated here in terms of ligand’s difference electronic density upon passing from vacuum to the enzyme-bound state.^47^ The change in the atomic partial charges *ΔQ*(*i*) is then derived from integration around each atom (Figure 5).

As expected, the polarization effects are more pronounced for the atomic species of the ligand which are involved in hydrogen bonds with the environment. The electronic density increased for the hydrogen bond acceptors of **2g**, while the hydrogen bond donor shows a decrease in the electronic density. On average, the total amount of redistributed charge within the ligand is 0.59 ± 0.05 *e*. The largest polarization effect is observed for the pyridone-O7 atom (−0.13 ± 0.03 *e*), possibly because of its strong interactions with the positively charged Lys53 residue and two water molecules. The tetrahydropyranyl-O1′ atom shows a decrease of −0.05 ± 0.02 *e* from the interactions with a water molecule, while the hydrogen bond interactions with the Met109 residue cause a charge shift of 0.03 ± 0.01 *e* and −0.03 ± 0.01 *e* for the amino HN and pyrimidine-N3 atoms, respectively.

**Figure 6:**
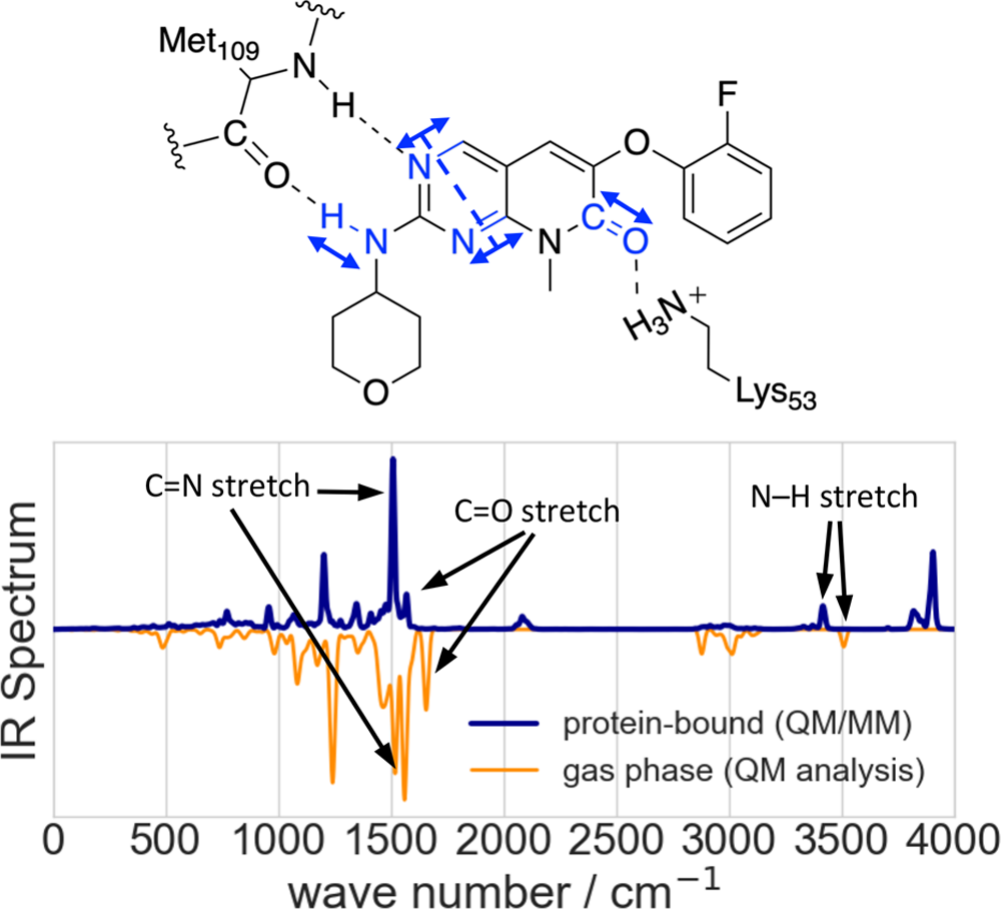
Vibrational analysis of the enzyme-bound ligand. Top panel: The scheme indicates important interactions of the ligand with the Met109 and Lys53 residues of p38α and vibrational modes of the ligand which are influenced by these interactions. Bottom panel: The IR spectrum of **2g** bound to the p38α MAPK enzyme is shown in blue. The spectrum was obtained from a 14.52 ps-long QM/MM MD simulation, and the intensity normalized to its strongest absorption band. It is compared to a normalized spectrum of **2g** (orange), which is determined from normal-mode analysis in the gas phase.

To investigate the effect of the enzyme environment on the ligand dynamics, we calculate the ligand’s infrared (IR) spectrum from the trajectory.^104^ Comparison is made with the spectrum of the ligand in the gas phase from a normal-mode analysis at the BLYP-D/def2-TZVP level of theory.^105−107^ Since differences in the basis sets, plane waves, and Gaussian-type orbitals are known to have a minor impact on the harmonic frequencies,^108^ the effect of ligand binding to the enzyme environment can be reasonably estimated from this comparison (Figure 6). We found that all the shifts of the chemical groups involved in H-bonds are consistent with those of previous studies,^109^ further validating our MiMiC-QM/MM method.

The fundamental mode with the highest frequency is observed at ≈3500 cm^–1^. It corresponds to the N–H stretching mode of the amino group of the ligand. A large shift of about −90 cm^–1^ is observed when passing from the vacuum to the enzyme-bound state, because the strong hydrogen bond with the carbonyl oxygen atom of residue Met109 weakens the N–H bond. The symmetric C=N stretching vibration within the pyrimidine moiety of **2g** is the most intense IR band and appears at ≈1560 cm^–1^ in the gas phase spectrum. This band is shifted by about −50 cm^–1^ in the enzyme-bound state because of the H-bond between the Met109 residue and the pyrimidine-N3 atom. The latter exhibits more structural flexibility that results in a less pronounced shift (see Table S2 in the Supporting Information). Finally, the C=O stretching vibration at ≈1655 cm^–1^ experiences a shift of about −85 cm^–1^ upon binding to the enzyme. This is caused by the interactions with water molecules and the Lys53 residue. The IR shift of protein/ligand H-bonds may be used to provide insight on the strength of the interactions between the two moieties. Under this assumption, we conclude that Met109 and Lys53 residues of the protein form particularly strong interactions with the ligand. [The simulation-based spectra calculations allow the observation of overtone and combination bands, which cannot be detected in the harmonic spectrum, as for example the spectral features at ≈3800 cm^–1^.]

In conclusion, our QM/MM MD simulations properly describe the dynamic impact of the enzyme environment on the ligand’s electronic structure and its internal dynamics – a prerequesite toward a balanced description of the unbinding process and, in turn, the accurate prediction of the ligand’s residence time. As a perspective of future work, one could gain insights on affinities through using dynamic undocking simulations^110^ at QM/MM accuracy starting from the bound state. In addition, it would be highly useful to compare electronic properties of ligands with different strengths to further assess the accuracy of QM/MM observables’ predictions.^111^

## Conclusions and Outlook

The MiMiC framework was built with two main goals in mind: to be able to use different QM and MM codes with great ease and to scale as well as possible.^68,69^ As showcased here and in previous work,^69^ MiMiC scales up to thousands of standard CPU cores and allows running up to several ps/day in a single QM/MM MD run. In particular, the extreme scalability at the B3LYP level indicates viability for an accurate description of enzymatic reactions when large computational resources are provided. Besides highlighting the efficient use of computational resources by the chosen QM layer (CPMD), these performances further demonstrate the effectiveness of a loose-coupling, multiple-program multiple-data paradigm for the development of extremely scalable first principle QM/MM interfaces.

As modern architectures make extensive use of heterogeneous nodes that combine multicore CPUs with GPUs,^55,112,113^ achieving exascale will require coupling GPU-ready MM and QM software able to scale on many (≈10^2|3^) such nodes. While a plethora of classical MD codes already exist that fully exploit GPUs,^114−117^ including GROMACS^71^ used in MiMiC, full implementation for these architectures is still an ongoing process for DFT codes,^61,63,65,66,118^ except for the TeraChem proprietary software.^56,57^ This is arguably the main reason why serious endeavors to port first principle QM/MM MD interfaces to GPUs are appearing only now in the literature.^119,120^

Strong scaling on heterogeneous nodes is actually the major challenge for molecular simulation. In force field based MD simulations, this is related to the relatively fixed size of the biological systems of interest^71^ and the intrinsic seriality of the time evolution integration algorithms. Attempts to overcome these limitations have leveraged on statistical mechanics-based ensemble methods,^121^ path sampling,^122^ and path-integral-like approaches,^123^ often combined with machine learning (ML) techniques.^124^ In DFT-based MD, only very recently scalability over thousands of GPUs has been achieved exploiting innovative linear scaling approaches and sparse algebra methods within an extended tight-binding scheme.^64^ These observations indicate the necessity to develop innovative algorithms and statistical mechanics based methods beyond standard MD approaches as a route toward exascale DFT QM/MM MD, an idea already explored in the context of semiempirical QM/MM simulations.^115^

As a very flexible multiscale framework, MiMiC is an excellent candidate to bring DFT QM/MM MD simulations to the exascale by coupling codes running on GPUs and exploiting massively parallel free energy methods. Massively parallel, pharmacologically oriented applications are envisaged in a not-too-far future.

Because of the cost associated with exascale calculations, we expect DFT QM/MM MD calculations to tremendously profit from the diffusion of ML techniques in molecular simulations.^125^ Indeed, hybrid ML/MM models enable the simulation of biological systems using an ML representation of a quantum mechanical potential at near QM/MM accuracy and at a fraction of the computational cost.^44,126−129^ These ML models work natively on GPUs, and because they normally rely on local interactions alone, they can be exceptionally scalable on distributed architectures.^72,73^ Furthermore, their training requires data sets generated through many single-point QM(/MM) calculations that are expensive but embarrassingly parallelizable. Finally, the recent introduction of ML-accelerated perturbative techniques provides an efficient and highly parallelizable way of recovering the accuracy of QM/MM potentials from simulations using cheaper methods (such as force fields or even ML/MM models) at the cost of only a few single-point energy and force QM/MM calculations.^46,130,131^ These methods, in combination with enhanced sampling approaches,^132^ promise to enable the QM/MM prediction of fundamental biophysical quantities such as drug–protein binding free energies or full free energy surfaces.

It is thus our hope that exascale DFT QM/MM MD simulations, combined with the power of ML approaches, will lead to a paradigm shift by bringing DFT-based QM/MM MD to the realm of drug discovery.

## Supporting Information Available

CPMD input files used to run the QM/MM simulations of the IDH1 and p38 systems, along with the force data from constrained MD and selected structures from the thermodynamics integration (ZIP)
Further computational details regarding the setup of the calculations; additional analyses of the classical MD simulations; QM/MM MD benchmark calculations for the **2g**/p38α system; X-ray, classical MD, and QM/MM MD structural data for the **2g**/p38α system (PDF)
