# Supplementary material for: Drug Design in the Exascale Era: A Perspective from Massively Parallel QM/MM Simulations
Source: J Chem Inf Model. 2023 Jun 15;63(12):3647–58. doi: 10.1021/acs.jcim.3c00557 (PMC10302481; doi:10.1021/acs.jcim.3c00557)
Supplement: Supplementary file 2 — ci3c00557_si_002.pdf [file ci3c00557_si_002.pdf]

# Supporting Information: Drug Design in the Exascale Era: A Perspective from Massively Parallel QM/MM Simulations

Bharath Raghavan,[†,‡] Mirko Paulikat,[†] Katya Ahmad,[†] Lara Callea,[¶] Andrea Rizzi,[†,§] Emiliano Ippoliti,[†] Davide Mandelli,[*,†] Laura Bonati,[¶] Marco De Vivo,[‖] and Paolo Carloni[*,†,⊥]

†*Computational Biomedicine, Institute of Advanced Simulations IAS-5/Institute for Neuroscience and Medicine INM-9, Forschungszentrum Jülich GmbH, Jülich 52428, Germany*

‡*Department of Physics, RWTH Aachen University, Aachen 52074, Germany*

¶*Department of Earth and Environmental Sciences, University of Milano-Bicocca, Piazza della Scienza 1, 20126 Milan, Italy*

§*Atomistic Simulations, Italian Institute of Technology, Genova 16163, Italy*

‖*Molecular Modelling and Drug Discovery, Italian Institute of Technology, Genova 16163, Italy*

⊥*Department of Physics and Universitätsklinikum, RWTH Aachen University, Aachen 52074, Germany*

E-mail: d.mandelli@fz-juelich.de; p.carloni@fz-juelich.de

# Investigation of an Enzymatic Reaction: The Case of Human Isocitrate Dehydrogenase-1

## Classical MD Simulations

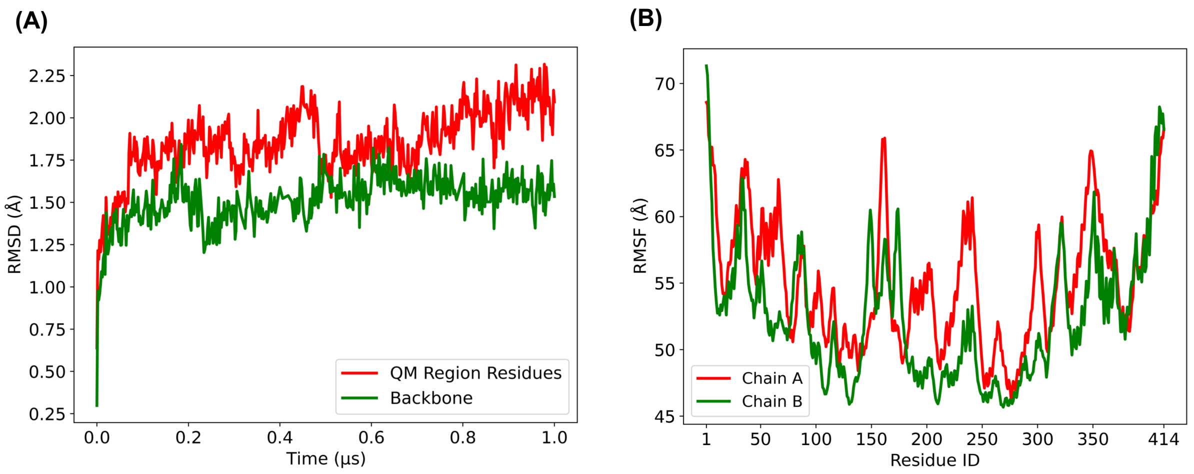


Figure S1: (A) Root Mean Squared Deviation of backbone and important QM residues in active sites of IDH1. (B) Root Mean Squared Fluctuation of subunit A and subunit B of IDH1.

The crystal structure of the human IDH1 enzyme (PDB ID: 1T0L)[S1] was solvated with TIP3P waters[S2] and the total charge of the simulation box was neutralized by adding $Na^+$ ions. The system (130,828 atoms in total) was then equilibriated using the GROMACS package[S3] by running a cumulative 1 µs of classical MD (cMD) using the Amber99sb*-ildn force field.[S4,S5] These simulations were performed on the CLAIX18 cluster of the RWTH Aachen University. The force field parameters for $NADP^+$ were obtained from a previous study[S6] and the parameters for isocitrate were generated using the Generalised Amber Force Field,[S7] and partial charges were parameterized using the RESP method at HF/6-31G* level of theory. This was done using the ANTECHAMBER software package and the python package ACPYPE.[S8,S9] A preliminary series of geometry optimization, NVT and NPT ensemble calculations were done to smoothly ramp up the temperature and pressure to standard conditions. Equilibrium simulations were carried out in the NPT ensemble with the temperature

and pressure maintained at 300K and 1 bar respectively.

Figure S1a shows the Root Mean Squared Deviation (RMSD) of the backbone carbons, and those of important residues (of subunit A) in the QM region during the course of the cMD simulations. The RMSD of backbone reaches a plateau after 0.2 µs, stabilizing around a value of ∼1.5 Å, showing minimal deviation from the crystal structure. The RMSD of the QM region residues stabilized at ∼2 Å. The analysis of the MD trajectory in the next section is done discarding the first 0.2 µs of equilibriation. Figure S1b shows the Root Mean Squared Fluctuations of the backbone carbons of the protein residues. Key residues in the active site, and involved in binding ligand, represent a local minimum in the fluctuations (like Arg100, Arg109, Arg132, Lys212, Asp252, Asp275, Asp279 and Glu306). This shows that these residues are conserved within the IDH1 enzyme and emphasizes their importance for protein function.

## Asp252$^B$-water-ICT Interaction

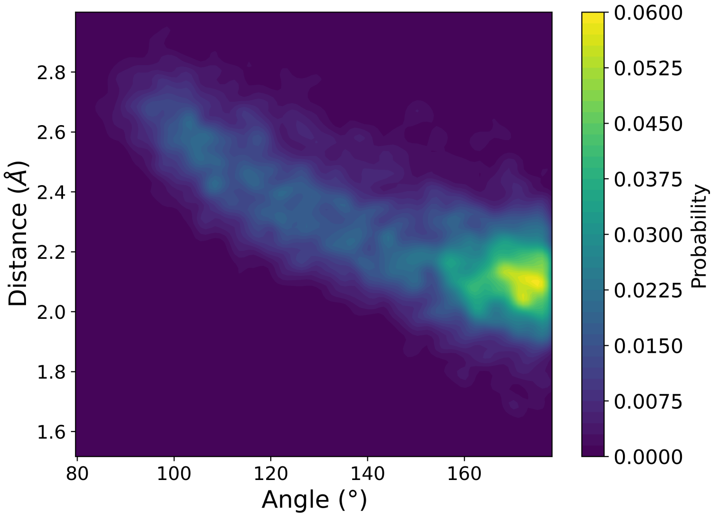


Figure S2: Histogram of $O_W$-$H_h$ distance versus $O_W$-$H_h$-$O_h$ angle for various points on the classical MD trajectory (refer to figure 3A in the main text for atom naming convention).

As discussed in the main text, we found a water molecule forming an H-bond with Asp252$^B$ and with α-alcohol of ICT in our classical MD simulations. We postulated that

this makes Asp252$^{B}$ well positioned to abstract a proton from the $C_\alpha$ hydroxyl of ICT, and initiated the IDH1 catalysis. The histogram of this interaction, i.e., the $O_W$-$H_h$ bonding distance versus the $O_W$-$H_h$-$O_h$ bonding angle (refer to figure 3A in the main text for atom naming convention) for various points across the classical MD simulations is shown in figure S2. We can see that the classical MD ensemble has a significant probability of existing in the space where the angle of bonding is between 170 and 180, and the distance is between 2 Å and 2.2 Å. This shows that the system spends significant time in the configuration where ICT and the water-Asp252$^{B}$ interact through a hydrogen bond. A point from this configuration is chosen as the initial coordinates for QM/MM.

## QM/MM Simulations

QM/MM MD was carried out using GROMACS 2020.3[S3] and CPMD 4.3[S10] interfaced with MiMiC 0.2.0[S11] (including the MiMiC Communication Library 2.0.1[S12] for server-client communication). A configuration from classical MD (discussed in the previous section) was used to convert MM inputs into MiMiC-QM/MM (CPMD and GROMACS) input files using the MiMiCPy package.[S13] All QM/MM simulations were performed on the JUWELS cluster of the Jülich Supercomputing Center.[S14] The total system consists of 130,828 atoms, of which 142 atoms were assigned into the QM region. It includes ICT, the nicotinamde ring of the NADP$^{+}$ cofactor, the magnesium ion and important residues from the active site (see figure 1a in the main text). The latter include : Arg100$^{A}$, Arg109$^{A}$, Lys212$^{B}$, Arg132$^{A}$, Tyr139$^{A}$, Asp275$^{A}$, Asp252$^{B}$ (along with the water molecule bound to it), Asp279$^{B}$ and two water molecules coordinated to $Mg^{2+}$.

Electrons were treated using DFT at the BLYP level.[S15] The core electrons were described using norm-conserving pseudopotentials[S16] and the valence electrons were treated explicitly. Open valences at the boundary of the QM-MM were treated with special monovalent pseudopotentials.[S17] A plane-wave basis set with a cutoff of 100 Ry was used to expand the wavefunctions and the Kohn-Sham equations were solved within an orthogonal

box of size 46.0 a.u. × 46.0 a.u. × 46.0 a.u. The rest of the system was treated with the Amber99sb*-ildn force field.[S4,S5]

Electrostatic interactions between the QM and MM subsystems were described using the Hamiltonian electrostatic coupling scheme of Laio *et al.*[S18] as implemented in the MiMiC library.[S19,S20] The short-range QM-MM electrostatic interactions were computed explicitly within a cutoff radius of 32 a.u., while the long-range interactions between the point charges of the MM region and the QM charge density were computed using a $5^{th}$ order multipole expansion of the QM electrostatic potential. Born-Oppenheimer MD was employed, with a timestep of 0.5 fs. Temperature was maintained around 300 K using a Nosé-Hoover chains thermostat.[S21]

Thermodynamic integration was performed in three step: (1) 18 QM/MM MD simulations (677 fs each) constraining $CV_1$, (2) a subsequent unconstrainted QM/MM MD of 677 fs, and (3) 19 QM/MM MD simulations (each of 1.4 ps) constraining $CV_2$. Statistical errors of the free energies were computed from 24 (in step 1) and 54 (in step 2) independent estimates of the potential of mean force obtained dividing the simulation into 50 chunks after discarding the first 95 fs.

## QM/MM MD Benchmarks

Figure S3 shows the strong scaling benchmarks performed using the BLYP[S15] functional, showing parallel efficiency > 70% up to 5,184 cores, achieving a performance of 5.4 ps/day.

Table S1 reports the configurations used for the B3LYP benchmarks of figure 1b of the main text. In our system, the size of the real space grid for the electronic density along the $x$-axis is 294, and the corresponding planes can be distributed across MPI tasks. CPMD allows for a second level of parallelization by defining the so called CP_groups.[S22] To achieve load balance, the total number of MPI tasks should be an integer divisor of 294×CP_groups.

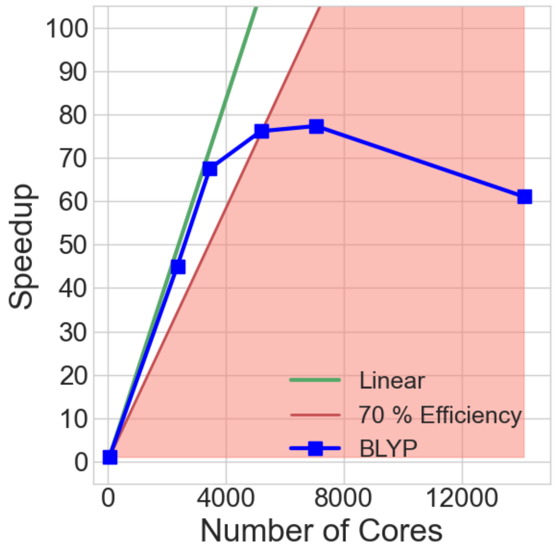


Figure S3: Strong scaling of MiMiC-based DFT QM/MM MD simulations at the BLYP level of theory of IDH1 as a function of the number of cores assigned to CPMD. In all simulations, we assigned one node (48 cores) to GROMACS. The speedup is provided in terms of the CPU time required for one MD step, normalized with respect to the reference run done on one node. All simulations have been performed on the JUWELS cluster.[S14]

# Investigation of Drug/Enzyme Interactions: The Case of 2g Binding to p38$\alpha$ Mitogen-activated Protein Kinase

## Methods

### Classical MD Simulations

The PDB structure and topology of the p38$\alpha$/**2g** Michaelis complex was obtained from the dataset published alongside the results of the high-throughput FEP workflow developed by Gapsys and coworkers.[S23] This dataset was employed in a previous publication of a similar approach using the Schrödinger software suite,[S24] which in turn used starting structures published in a large-scale preclinical study of human p38$\alpha$ MAP kinase inhibitors.[S25] The topology used Amber99sb*-ildn force field parameters for the protein[S4,S5] and GAFF parameters for the ligand.[S7] The ligand used in the simulations described here, 6-(2-fluorophenoxy)-8-methyl-2-(tetrahydro-2*H*-pyran-4-ylamino)pyrido[2,3-*d*]pyrimidin-7(8*H*)-one was given the

Table S1: The configurations used in the B3LYP QM/MM MD benchmarks of the IDH1 system reported in figure 1 of the main text (only the nodes utilized by CPMD are reported). We report the number of nodes used, the total number of cores (there are 48 cores/node on JUWELS), the number of MPI tasks per node, the total number of MPI tasks, the number of CPMD CP_groups,[S22] and the expected number of FFT planes/task.

| Nodes | Cores | Task/Node | Tasks | CP Groups | # Planes/Task | Efficiency (%) |
|---|---|---|---|---|---|---|
| 7 | 336 | 6 | 42 | 1 | 7 | 100 |
| 147 | 7,056 | 6 | 882 | 6 | 2 | 83 |
| 294 | 14,112 | 6 | 1764 | 12 | 2 | 78 |
| 588 | 28,224 | 4 | 2352 | 24 | 3 | 72 |
| 882 | 42,336 | 6 | 5302 | 36 | 2 | 74 |
| 1176 | 56,448 | 6 | 7056 | 48 | 2 | 60 |
| 1470 | 70,560 | 6 | 8820 | 60 | 2 | 65 |
| 1764 | 84,672 | 6 | 10584 | 72 | 2 | 69 |

identifier **2g** in a previous studies.[S25] It is an analogue of the **2a** ligand, specifically 6-(2,4-difluorophenoxy)-8-methyl-2-(tetrahydro-2*H*-pyran-4-ylamino)pyrido[2,3-*d*]pyrimidin-7(8*H*)-one (see figure 4 in main text). The X-ray crystal structure of the human p38$\alpha$ MAP kinase in complex with **2a** (PDB code: 3FLN) was used as the basis for the construction of the p38$\alpha$/**2g** complex in the previous study,[S24] which was achieved by modifying the ligand using the Schrödinger Protein Preparation Wizard.[S26]

The initial PDB structure of the protein-ligand (**2g**) complex from the dataset was stripped of the pre-existing water and ions and solvated in a 12 nm $\times$ 12 nm$\times$ 12 nm cubic box of TIP3P water[S2] and neutralized with $Na^+$ and $Cl^-$ ions[S27] corresponding to a concentration of 0.033 mol dm$^{-3}$ of NaCl using GROMACS solvate and genion modules.[S3] The final system consisted of 169550 atoms, and was subjected to 3000 steps of energy minimization through the steepest descent algorithm implemented in GROMACS 2020.4.[S3] All further equilibration and production simulations were conducted with GROMACS, using a 2 fs time step, the P-LINCS algorithm for constraints,[S28] the Bussi velocity-rescaling thermostat with a time constant of 0.1 ps,[S29] and PME electrostatics with a 1 nm cutoff and a Fourier grid spacing of 0.12 nm.[S30] After energy minimization, the system was equilibrated for 1 ns in the NVT ensemble with the temperature maintained at 300 K. This was followed

by 1 ns of NPT equilibration at 1 bar with the Berendsen barostat with a time constant of 2 ps for the initial relaxation of the box volume,[S31] followed by a further 150 ns of equilibration at 1 bar with the Parrinello-Rahman barostat,[S32] with a time constant of 2 fs. The duration of the equilibration phase was sufficient for the RMSD of the protein to stably converge at $0.208 \pm 0.027$ nm. An ensemble of 10 production mode MD simulations of 50 ns duration was then conducted.

## QM/MM MD Simulations

The initial QM/MM configuration was taken from the last snapshot of a production run of the classical MD simulation. The MiMiC-QM/MM input files were generated from the classical MD files using MiMiCPy.[S13] The ligand (**2g**) was treated at the QM level (46 atoms) while the rest was described at the MM level. The MM subsystem was described by the same force fields as in the classical MD simulations. The QM problem was solved within the plane wave-pseudopotential density functional theory (PW-PP DFT) framework. The BLYP functional was employed,[S15] including Grimme's dispersion correction.[S33] A 90 Ry energy cutoff was used for the PW basis set and Troullier-Martins PPs described the valence-core electron interactions.[S16] The QM system, which was placed in a rectangular unit cell with dimensions of 13.02 Å× 15.62 Å × 20.83 Å, was decoupled from the periodic images with the Martyna-Tuckerman solver. The QM and MM subsystems were electrostatically coupled,[S18] as implemented in the MiMiC interface.[S19,S20] MM atoms within a cutoff of 26.5 Å were explicitly considered, while a multipole expansion up to 5th order was used for long-range interactions. Born-Oppenheimer molecular dynamics were run with a time step of 0.48 fs. At first, the system was relaxed through a 0.48 ps simulation, in which the atoms velocities were damped by a factor of 0.99 in each step from an initial Maxwell-Boltzmann velocity distribution of 300 K. Then, the system was re-heated to 300 K in a 9.68 ps simulation using the Berendsen thermostat (coupling strength of 5000 au) and a linear heating rate of 0.04 K fs$^{-1}$.[S31] A 9.68 ps NVT simulation at 300 K followed to equilibrate the system

using the Nosé-Hoover thermostat (coupling frequency of 3000 $cm^{-1}$).[S21] Finally, the system underwent further 100 ps QM/MM simulations at 300 K. The trajectory was stored every 0.048 ps for structural analysis.

## Electronic Polarization

The electronic density of the ligand (both in vacuo and in the bound state) was calculated every 5 ps for 20 equidistant QM/MM snapshots, using the same settings as outlined above. The change in electron density was computed as:

$$\Delta\rho = \rho_{\text{lig}}^{\text{complex}} - \rho_{\text{lig}}^{\text{vacuo}} \tag{1}$$

The density of the complex is obtained by performing the calculation in the presence of the electric field of the surrounding protein and the aqueous solvent. By integrating the $\Delta\rho$, the change in the atomic charges for each ligand atom ($i$) was monitored:

$$\Delta Q(i) = \int \Delta\rho(\mathbf{r})\mathrm{d}\mathbf{r} \tag{2}$$

The integral is solved numerically over the grid points within the Voronoi partition of atom $i$ using the code from Ref. S34. An estimation of the charge redistribution upon ligand binding is given by:

$$\Delta Q_{\text{pol}} = |\Delta Q(+)| + |\Delta Q(-)| \tag{3}$$

where:

$$\Delta Q(+) = \sum_i \Delta Q(i), \quad i \in \{\Delta Q(i) > 0\} \tag{4}$$

$$\Delta Q(-) = \sum_i \Delta Q(i), \quad i \in \{\Delta Q(i) < 0\} \tag{5}$$

### Vibrational Spectroscopy

The QM/MM simulations of the **2g** ligand in the p38$\alpha$ enzyme binding pocket were continued for 14.52 ps at 300 K using the same settings as described above. Additionally, the dipole moment $\boldsymbol{\mu}$ of the ligand was calculated every 2.42 fs to collect the dipole moment series. The intensity of the infrared (IR) spectrum was then derived from the Fourier transform of the dipole moment autocorrelation function (ACF):[S35]

$$I(\omega) \propto \int \mathrm{d}t \exp^{-i\omega t} \langle \boldsymbol{\mu}(0) \cdot \boldsymbol{\mu}(t) \rangle \tag{6}$$

where $\omega$ and $t$ are the angular frequency and time, respectively. The dipole moment ACF is given by

$$\langle \boldsymbol{\mu}(0) \cdot \boldsymbol{\mu}(t) \rangle = \sum_{i}^{N} = \frac{t}{N - \frac{t_i}{\Delta t + 1}} \boldsymbol{\mu}(0) \cdot \boldsymbol{\mu}(t_i + t) \tag{7}$$

Here, $\Delta t$ is the integration timestep (2.42 fs) and $N$ is the total number of steps (30,000). The TRAVIS program package was used to process the time series data.[S36]

A reference spectrum of ligand **2g** was calculated in the gas phase from quantum chemistry calculations. The initial structure from the QM/MM setup was optimized at the BLYP-D/def2-TZVP level of theory.[S37–S39] The resolution-of-the-identity method was employed to speedup the calculation of the Coulomb integrals. Then, a normal mode analysis was carried out to obtain the IR spectrum in the double harmonic approximation. The quantum chemical calculations were done with the ORCA 4.0.1 program package.[S40]

## QM/MM MD Benchmarks

In a first set of benchmark calculations ($i$), we applied the same system settings as described above. Three different plane wave cutoffs were used: a small (75 Ry) and a medium-sized (90 Ry) value, typically applied in QM/MM simulations, and a large 120 Ry energy cutoff.

The heated structure served as initial configurations and underwent 0.48 ps NVT simulations at 300 K for each cutoff value. Then, the system underwent 20 BO-MD steps, for which the last 10 steps were used for analysis. In a second set of benchmark calculations (*ii*), we applied a cubic QM box size with edge length of 21.16 Å and a plane wave cutoff of 90 Ry. Otherwise, the protocol was the same as described above. Beside the BLYP functional, we also show the scaling behavior for the B3LYP functional. Hereby, we evaluated the scaling based on only one MD step due to the increasing computational cost of these simulations. All calculations were carried out on the CPU module of the JUWELS cluster, consisting of nodes with 2 $\times$ Intel Xeon Platinum 8168 CPU (48 cores per node). The timings are normalized with respect to the ones on one node. Hereby, we explored different settings for OpenMP threads, varying within $\{1,2,4,6,8,12,16,24\}$, while the number of MPI tasks were conveniently adjusted. In the case of the B3LYP functional, we also benchmarked the number of processor groups as implemented in CPMD. The results are shown in figure S4.

(*i*) The QM/MM calculations efficiently scale up to 384 cores (figure S4A). Apparently, the scalability is increased when larger energy cutoffs, and in turn a larger number of plane waves, are employed. Regarding the performance of the simulations, the utility of our code is clearly established. We are able to simulate 21−23 ps/day on 384 cores with a reasonable size of the plane wave expansion. This efficient sampling already allows QM/MM studies of enzyme-ligand systems in the sub-ns regime, just requiring a few weeks of total simulation time.

(*ii*) The scalability of the BLYP simulations is strongly increased, when a cubic QM box is employed (figure S4B). Importantly, such a setup would be required to take into account the conformational flexibility of the ligand upon the unbinding process. The calculations efficiently scale up to $\approx 1,000$ cores, while the performance with $\approx$ 20 ps/day is almost retained in comparison to the calculations in (*i*). Benchmarks at the B3LYP level shows a strong scaling up to 12,288 cores with an efficiency of $\approx$ 70% (figure S4C), reaching a performance of 4.8 ps/day. The extreme scalability observed in our IDH enzyme simulations

is not fully reached through the lower number of QM atoms (46 compared to 156 atoms) and smaller QM box sizes. However, a similar scaling behavior is expected as soon as the QM region of the p38$\alpha$/**2g** system would be increased by inclusion of protein residues.

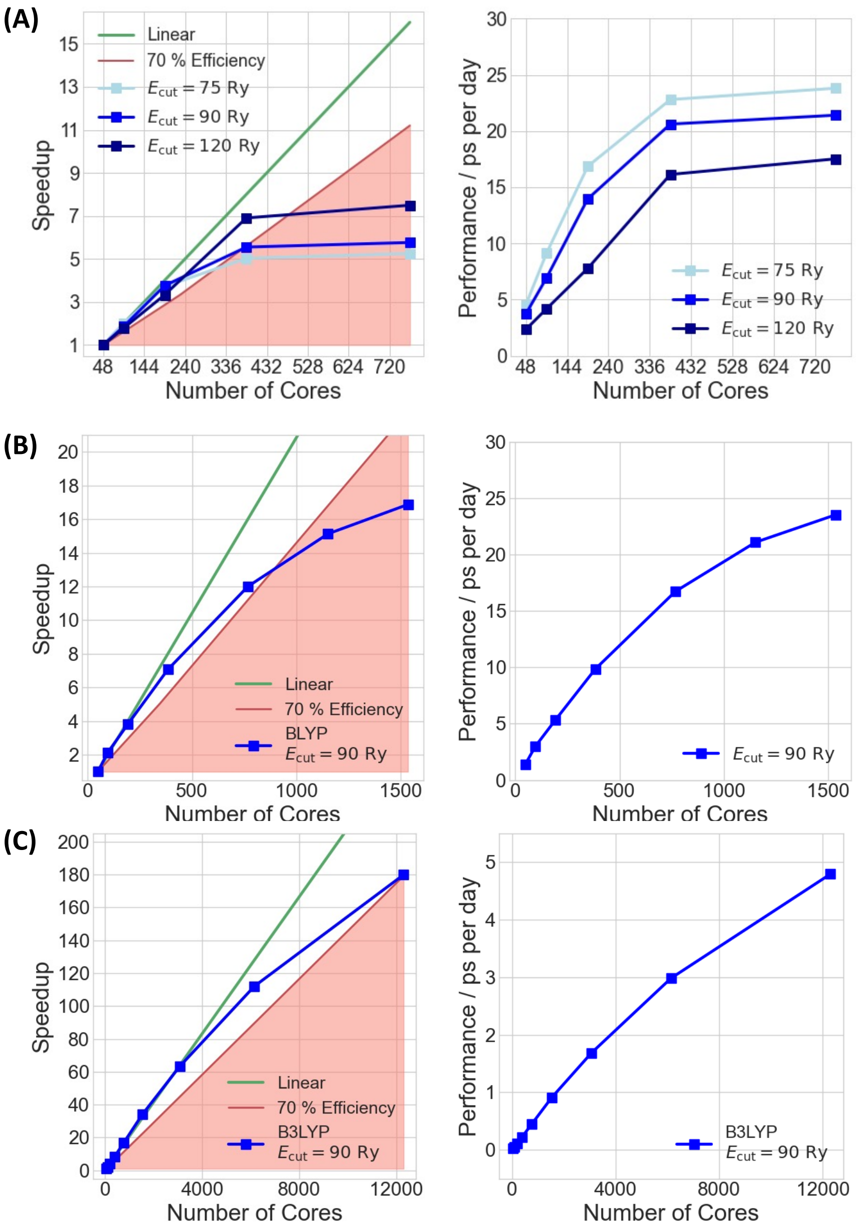


Figure S4: Scaling and performance behaviors of MiMiC-based DFT QM/MM-MD on the CPU module of the JUWELS cluster for the p38$\alpha$/**2g** complex. The speedup is provided in terms of the average CPU times required for ten MD steps (BLYP) or one MD step (B3LYP), normalized with respect to the reference runs on one node (48 cores). In all simulations, we additionally allocated one node for GROMACS. (A) BLYP QM/MM MD scaling at three plane wave cutoffs for an orthorhombic box of dimension 13.02 Å×15.62 Å×20.83 Å, applied in the calculations of the complex in this study (see main text). (B/C) Demonstration of BLYP/B3LYP QM/MM MD scaling for a cubic box with edge length of 21.16 Å, being suitable to take into account the conformational flexibility of the ligand in the unbinding process.

# Supplementary Results

## Classical MD Simulations

The solvated p38$\alpha$/**2g** complex was first subject to 500 ns classical MD simulations at 300 K. The overall binding motif of the complex is retained in comparison to the crystal structure of its ligand analogue (see Table S2). However, water mediated changes in the binding interactions are observed at the solvent exposed side of the complex. In particular, the salt bridge between residue Lys53 and Glu71 is interrupted by water insertion in 40 % of the sampled configurations (figure S5, figure 4 in the main text). Thereby, the positively charged side chain of the Lys53 residue gets close to the pyridone oxygen atom of the ligand with a $O_{2g} \cdots H_{Lys53}$ distance lower than 4 Å in 21 % of the sampled configurations, giving the potential to direct binding to the ligand (figure S5, figure 4 in the main text). Moreover, there is a conformational change in the Asp112 residue to form a water mediated interaction with the tetrahydropyranyl oxygen atom (figure 4 in the main text). The structural binding motif is used to study the p38$\alpha$/**2g** complex by MiMiC-based QM/MM MD simulations.

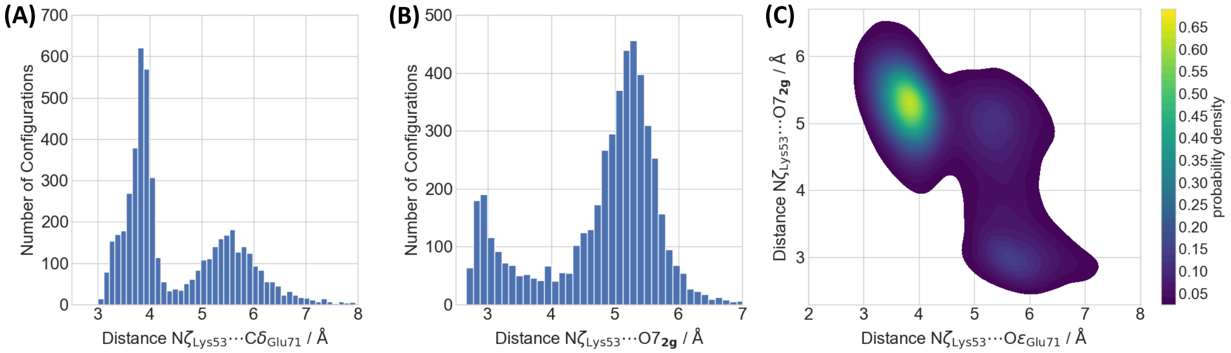


Figure S5: Structural analysis of the ligand **2g** – Lys53 – Glu71 interactions, showing the histograms of the (A) $N\zeta_{Lys53}-C\delta_{Glu71}$ and (B) $N\zeta_{Lys53}-O7_{\mathbf{2g}}$ distances, and (C) both distances in a normalized 2D-histogram. 5,000 equidistant structures were taken from the 500 ns classical MD simulations for the analysis.

**Structural Data from Classical MD and QM/MM MD Simulations**

Table S2: Averaged structural data of the **2g**/p38$\alpha$ complex from the 500 ns classical MD simulation and the 100 ps QM/MM MD simulation in comparison to the crystal structure of the **2a**/p38$\alpha$ complex (PDB code: 3FLN). The atom naming convention of the ligand is defined in figure 5 in the main text. All data are given in Å, except the last two entries specifying the H-bond angles in degrees.

| Residue | Atom(s)@Residue | Atom(s)@Ligand | Crystal Structure | Classical MD | QM/MM MD |
|---|---|---|---|---|---|
| Val38 | C$\gamma$1 | C2” | 4.82 | 4.86±0.46 | 5.36±0.32 |
| Val38 | C$\gamma$2 | C7 | 3.78 | 4.19±0.37 | 4.55±0.39 |
| Ala51 | C$\beta$ | C4 | 3.42 | 3.64±0.28 | 3.53±0.21 |
| Ile84 | C$\gamma$2 | C6” | 4.04 | 4.07±0.54 | 3.86±0.27 |
| Leu86 | C$\delta$ | C4” | 4.66 | 4.87±1.19 | 4.23±0.37 |
| Leu104 | C$\beta$ | C4” | 3.81 | 3.80±0.26 | 3.61±0.17 |
| Thr106 | C$\gamma$ | C3” | 3.51 | 3.54±0.20 | 3.51±0.16 |
| Thr106 | C$\gamma$ | C5 | 3.85 | 3.96±0.22 | 3.97±0.18 |
| Leu167 | C$\delta$ | N8 | 3.79 | 4.22±0.55 | 4.01±0.44 |
| Met109 | N | N3 | 3.03 | 3.12±0.16 | 3.14±0.14 |
| Met109 | H | N3 | — | 2.18±0.19 | 2.21±0.17 |
| Met109 | O | NH | 2.86 | 3.00±0.19 | 2.87±0.12 |
| Met109 | O | HN | — | 2.05±0.20 | 1.89±0.13 |
| Lys53 | N$\zeta$ | O7 | 5.20 | 4.78±0.94 | 2.88±0.13 |
| Lys53 | H$\zeta$ | O7 | — | — | 1.98±0.18 |
| Met109 | N−H | N3 | — | 157°±12° | 155°±12° |
| Met109 | O | H−N | — | 158°± 9° | 161°± 8° |

# References


(S1) Xu, X.; Zhao, J.; Xu, Z.; Peng, B.; Huang, Q.; Arnold, E.; Ding, J. Structures of human cytosolic NADP-dependent isocitrate dehydrogenase reveal a novel self-regulatory mechanism of activity. *J. Biol. Chem.* **2004**, *279*, 33946–33957.

(S2) Mark, P.; Nilsson, L. Structure and dynamics of the TIP3P, SPC, and SPC/E water models at 298 K. *J. Phys. Chem. A* **2001**, *105*.

(S3) Abraham, M. J.; Murtola, T.; Schulz, R.; Páll, S.; Smith, J. C.; Hess, B.; Lindah, E. GROMACS: High performance molecular simulations through multi-level parallelism from laptops to supercomputers. *SoftwareX* **2015**, *1-2*, 19–25.

(S4) Best, R. B.; Hummer, G. Optimized Molecular Dynamics Force Fields Applied to the HelixCoil Transition of Polypeptides. *J. Phys. Chem. B* **2009**, *113*, 9004–9015.

(S5) Lindorff-Larsen, K.; Piana, S.; Palmo, K.; Maragakis, P.; Klepeis, J. L.; Dror, R. O.; Shaw, D. E. Improved side-chain torsion potentials for the Amber ff99SB protein force field. *Proteins* **2010**, *78*, 1950–1958.

(S6) Holmberg, N.; Ryde, U.; Bülow, L. Redesign of the coenzyme specificity in l-Lactate dehydrogenase from Bacillus stearothermophilus using site-directed mutagenesis and media engineering. *Protein Eng. Des. Sel.* **1999**, *12*, 851–856.

(S7) Wang, J.; Wolf, R. M.; Caldwell, J. W.; Kollman, P. A.; Case, D. A. Development and testing of a general amber force field. *J. Comput. Chem.* **2004**, *25*, 1157–1174.

(S8) Wang, J.; Wang, W.; Kollman, P. A.; Case, D. A. Automatic atom type and bond type perception in molecular mechanical calculations. *J. Mol. Graphics Modell.* **2006**, *25*, 247–260.

(S9) Silva, A. W. S. D.; Vranken, W. F. ACPYPE - AnteChamber PYthon Parser interfacE. *BMC Res. Notes* **2012**, *5*, 1–8.

(S10) Hutter, J.; Alavi, A.; Deutsch, T.; Bernasconi, M.; Goedecker, S.; Marx, D.; Tuckerman, M.; Parrinello, M. CPMD, Copyright IBM Corp 1990-2022, Copyright MPI für Festkörperforschung Stuttgart 1997-2001. `http://www.cpmd.org/`.

(S11) Olsen, J. M. H.; Bolnykh, V.; Meloni, S.; Ippoliti, E.; Carloni, P.; Rothlisberger, U. MiMiC: A Framework for Multiscale Modeling in Computational Chemistry (v0.2.0). 2022.

(S12) Bolnykh, V.; Olsen, J. M. H.; Meloni, S.; Ippoliti, E.; Carloni, P.; Rothlisberger, U. MiMiC Communication Library (v2.0.1). 2022.

(S13) Raghavan, B.; Schackert, F. K.; Levy, A.; Johnson, S. K.; Ippoliti, E.; Mandelli, D.; Olsen, J. M. H.; Rothlisberger, U.; Carloni, P. MiMiCPy: An Efficient Toolkit for MiMiC-Based QM/MM Simulations. *J. Chem. Inf. Model.* **2023**,

(S14) Alvarez, D. JUWELS Cluster and Booster: Exascale Pathfinder with Modular Supercomputing Architecture at Juelich Supercomputing Centre. *Journal of large-scale research facilities JLSRF* **2021**, *7*.

(S15) Becke, A. D. Density-functional thermochemistry. I. The effect of the exchange-only gradient correction. *J. Chem. Phys.* **1992**, *96*, 2155–2160.

(S16) Troullier, N.; Martins, J. L. Efficient pseudopotentials for plane-wave calculations. *Phys. Rev. B* **1991**, *43*, 1993–2006.

(S17) Von Lilienfeld, O. A.; Tavernelli, I.; Rothlisberger, U.; Sebastiani, D. Variational optimization of effective atom centered potentials for molecular properties. *J. Chem. Phys.* **2004**, *122*, 014113.

(S18) Laio, A.; VandeVondele, J.; Rothlisberger, U. A Hamiltonian electrostatic coupling scheme for hybrid Car–Parrinello molecular dynamics simulations. *J. Chem. Phys.* **2002**, *116*, 6941.

(S19) Olsen, J. M. H.; Bolnykh, V.; Meloni, S.; Ippoliti, E.; Bircher, M. P.; Carloni, P.; Rothlisberger, U. MiMiC: A Novel Framework for Multiscale Modeling in Computational Chemistry. *J. Chem. Theory Comput.* **2019**, *15*, 3810–3823.

(S20) Bolnykh, V.; Olsen, J. M. H.; Meloni, S.; Bircher, M. P.; Ippoliti, E.; Carloni, P.; Rothlisberger, U. Extreme Scalability of DFT-Based QM/MM MD Simulations Using MiMiC. *J. Chem. Theory Comput.* **2019**, *15*, 5601–5613.

(S21) Nosé, S. A unified formulation of the constant temperature molecular dynamics methods. *J. Chem. Phys.* **1998**, *81*, 511.

(S22) Weber, V.; Bekas, C.; Laino, T.; Curioni, A.; Bertsch, A.; Futral, S. Shedding Light on Lithium/Air Batteries Using Millions of Threads on the BG/Q Supercomputer. 2014 IEEE 28th International Parallel and Distributed Processing Symposium. 2014; pp 735–744.

(S23) Gapsys, V.; Pérez-Benito, L.; Aldeghi, M.; Seeliger, D.; van Vlijmen, H.; Tresadern, G.; de Groot, B. L. Large scale relative protein ligand binding affinities using non-equilibrium alchemy. *Chem. Sci.* **2020**, *11*, 1140–1152.

(S24) Wang, L.; Wu, Y.; Deng, Y.; Kim, B.; Pierce, L.; Krilov, G.; Lupyan, D.; Robinson, S.; Dahlgren, M. K.; Greenwood, J.; Romero, D. L.; Masse, C.; Knight, J. L.; Steinbrecher, T.; Beuming, T.; Damm, W.; Harder, E.; Sherman, W.; Brewer, M.; Wester, R.; Murcko, M.; Frye, L.; Farid, R.; Lin, T.; Mobley, D. L.; Jorgensen, W. L.; Berne, B. J.; Friesner, R. A.; Abel, R. Accurate and Reliable Prediction of Relative Ligand Binding Potency in Prospective Drug Discovery by Way of a Modern Free-Energy Calculation Protocol and Force Field. *J. Am. Chem. Soc.* **2015**, *137*, 2695–2703.

(S25) Goldstein, D. M.; Soth, M.; Gabriel, T.; Dewdney, N.; Kuglstatter, A.; Arzeno, H.; Chen, J.; Bingenheimer, W.; Dalrymple, S. A.; Dunn, J.; Farrell, R.; Frauchiger, S.; Fargue, J. L.; Ghate, M.; Graves, B.; Hill, R. J.; Li, F.; Litman, R.; Loe, B.; McIntosh, J.; McWeeney, D.; Papp, E.; Park, J.; Reese, H. F.; Roberts, R. T.; Rotstein, D.; Pablo, B. S.; Sarma, K.; Stahl, M.; Sung, M.-L.; Suttman, R. T.; Sjogren, E. B.; Tan, Y.; Trejo, A.; Welch, M.; Weller, P.; Wong, B. R.; Zecic, H. Discovery of 6-(2,4-Difluorophenoxy)-2-[3-hydroxy-1-(2-hydroxyethyl)propylamino]-8-methyl-8 *H* -pyrido[2,3-*d*]pyrimidin-7-one (Pamapimod) and 6-(2,4-Difluorophenoxy)-8-methyl-2-(tetrahydro-2 *H* -pyran-4-ylamino)pyrido[2,3- *d* ]pyrimidin-7(8 *H* )-one (R1487) as Orally Bioavailable and Highly Selective Inhibitors of p38α Mitogen-Activated Protein Kinase. *J. Med. Chem.* **2011**, *54*, 2255–2265.

(S26) Sastry, G. M.; Adzhigirey, M.; Day, T.; Annabhimoju, R.; Sherman, W. Protein and ligand preparation: parameters, protocols, and influence on virtual screening enrichments. *J. Comput.-Aided Mol. Des.* **2013**, *27*, 221–234.

(S27) Joung, I. S.; Cheatham, T. E. Determination of Alkali and Halide Monovalent Ion Parameters for Use in Explicitly Solvated Biomolecular Simulations. *J. Phys. Chem. B* **2008**, *112*, 9020–9041.

(S28) Hess, B.; Bekker, H.; Berendsen, H. J. C.; Fraaije, J. G. E. M. LINCS: A linear constraint solver for molecular simulations. *J. Comput. Chem.* **1997**, *18*, 1463–1472.

(S29) Bussi, G.; Donadio, D.; Parrinello, M. Canonical sampling through velocity rescaling. *J. Chem. Phys.* **2007**, *126*, 014101.

(S30) Essmann, U.; Perera, L.; Berkowitz, M. L.; Darden, T.; Lee, H.; Pedersen, L. G. A smooth particle mesh Ewald method. *J. Chem. Phys.* **1995**, *103*, 8577–8593.

(S31) Berendsen, H. J. C.; Postma, J. P. M.; van Gunsteren, W. F.; DiNola, A.; Haak, J. R. Molecular dynamics with coupling to an external bath. *J. Chem. Phys.* **1984**, *81*, 3684–3690.

(S32) Parrinello, M.; Rahman, A. Polymorphic transitions in single crystals: A new molecular dynamics method. *J. Appl. Phys.* **1981**, *52*, 7182–7190.

(S33) Grimme, S. Semiempirical GGA-type density functional constructed with a long-range dispersion correction. *J. Comput. Chem.* **2006**, *27*, 1787–1799.

(S34) Capelli, R.; Lyu, W.; Bolnykh, V.; Meloni, S.; Olsen, J. M. H.; Rothlisberger, U.; Parrinello, M.; Carloni, P. Accuracy of Molecular Simulation-Based Predictions of k off Values: A Metadynamics Study. *J. Phys. Chem. Lett.* **2020**, *11*, 6373–6381.

(S35) Morzan, U. N.; de Armiño, D. J. A.; Foglia, N. O.; Ramírez, F.; Lebrero, M. C. G.;

Scherlis, D. A.; Estrin, D. A. Spectroscopy in Complex Environments from QM–MM Simulations. *Chem. Rev.* **2018**, *118*, 4071–4113.

(S36) Brehm, M.; Thomas, M.; Gehrke, S.; Kirchner, B. TRAVIS—A free analyzer for trajectories from molecular simulation. *J. Chem. Phys.* **2020**, *152*, 164105.

(S37) Becke, A. D. Density-functional thermochemistry. II. The effect of the Perdew–Wang generalized-gradient correlation correction. *J. Chem. Phys.* **1998**, *97*, 9173.

(S38) Grimme, S.; Antony, J.; Ehrlich, S.; Krieg, H. A consistent and accurate ab initio parametrization of density functional dispersion correction (DFT-D) for the 94 elements H-Pu. *J. Chem. Phys.* **2010**, *132*.

(S39) Weigend, F.; Ahlrichs, R. Balanced basis sets of split valence, triple zeta valence and quadruple zeta valence quality for H to Rn: Design and assessment of accuracy. *Phys. Chem. Chem. Phys.* **2005**, *7*, 3297.

(S40) Neese, F. The ORCA program system. *Wiley Interdiscip. Rev. Comput. Mol. Sci.* **2012**, *2*, 73–78.
